# Quaternary ammonium-based coating of textiles is effective against bacteria and viruses with a low risk to human health

**DOI:** 10.1038/s41598-023-47707-3

**Published:** 2023-11-23

**Authors:** Philipp Meier, Pietro Clement, Stefanie Altenried, Giacomo Reina, Qun Ren, Roland Züst, Olivier Enger, Francis Choi, Nikolaus Nestle, Ted Deisenroth, Peter Neubauer, Peter Wick

**Affiliations:** 1 Particles-Biology Interactions Laboratory, Empa-Swiss Federal Laboratories for Materials Science and Technology, 9014 St. Gallen, Switzerland; 2 Biointerfaces Laboratory, Empa-Swiss Federal Laboratories for Materials Science and Technology, 9014 St. Gallen, Switzerland; 3 Federal Office for Civil Protection FOCP, Spiez Laboratory, 3700 Spiez, Switzerland; 4 Technology Scouting & Incubation, BASF Schweiz AG, 4005 Basel, Switzerland; 5 BASF Corporation, 1609 Biddle Avenue, Wyandotte, MI 48192 USA; 6 BASF SE, Carl-Bosch-Strasse 38, 67056 Ludwigshafen am Rhein, Germany; 7 Formulation Research, BASF Corporation, 500 White Plains Road, Tarrytown, NY 10591 USA; 8 Chair of Bioprocess Engineering, Institute of Biotechnology, TU Berlin, 13355 Berlin, Germany

**Keywords:** Bacterial infection, Viral infection, Cell-particle interactions, Medical toxicology, Pathogens, Virology

## Abstract

While the global healthcare system is slowly recovering from the COVID-19 pandemic, new multi-drug-resistant pathogens are emerging as the next threat. To tackle these challenges there is a need for safe and sustainable antiviral and antibacterial functionalized materials. Here we develop an 'easy-to-apply' procedure for the surface functionalization of textiles, rendering them antiviral and antibacterial and assessing the performance of these textiles. A metal-free quaternary ammonium-based coating was applied homogeneously and non-covalently to hospital curtains. Abrasion, durability testing, and aging resulted in little change in the performance of the treated textile. Additionally, qualitative and quantitative antibacterial assays on *Staphylococcus aureus*, *Pseudomonas aeruginosa,* and *Acinetobacter baumanii* revealed excellent antibacterial activity with a CFU reduction of 98–100% within only 4 h of exposure. The treated curtain was aged 6 months before testing. Similarly, the antiviral activity tested according to ISO-18184 with murine hepatitis virus (MHV) showed > 99% viral reduction with the functionalized curtain. Also, the released active compounds of the coating 24 ± 5 µg mL^−1^ revealed no acute in vitro skin toxicity (IC_50_: 95 µg mL^−1^) and skin sensitization. This study emphasizes the potential of safe and sustainable metal-free textile coatings for the rapid antiviral and antibacterial functionalization of textiles.

## Introduction

In the coming years, the global healthcare system will face major challenges in the field of multidrug-resistant pathogens^1,2^. Predictive statistical models associated 4.95 million deaths yearly in connection to bacterial antimicrobial multi-resistances (AMR) and 1.27 million deaths solely attributed to AMR in the year 2019 on a global scale^2^. The most relevant pathogens that cause AMR are *Escherichia coli*, *Staphylococcus aureus*, *Klebsiella pneumoniae*, *Streptococcus pneumoniae*, *Acinetobacter baumannii*, and *Pseudomonas aeruginosa*^1,2^. Additionally, new strains of viral pathogens (influenza, COVID-19, and monkeypox) are a global threat. The coronavirus SARS-CoV-2 pandemic caused 528 million COVID-19 cases worldwide and resulted in 6.3 million deaths since it first emerged in early 2020^3^. Due to factors such as a high transmission rate, superspreading events, and lack of immunological resistance, the SARS-CoV-2 pandemic showed the highest global number of deaths in the past 200 years^4^. This emphasizes the risks of potential future pandemics caused by viral diseases such as SARS, Influenza, MERS, Avian influenza, Dengue, Zika, Chikungunya, Yellow fever, West Nile encephalitis, Japanese encephalitis, Ebola, and Monkeypox^3–5^.

During a pandemic, the production and delivery of personal protection equipment and other antiviral and antibacterial treated materials become critical to rapidly interrupt the propagation of infectious microbes^6^. Textiles without functionalization can only trap microbial-containing aerosols either within their filter capacity range or via electrostatic charges^6,7^. A fast response to microbial threats with efficient personal protective equipment is necessary to break the transmission chain. Technologies for antiviral and antibacterial surface functionalization are well established and gained more attention during the pandemic years^6,8^. Seidi et al. (2021) classified four modes of action: (i) enhanced antimicrobial and self-disinfectant properties by incorporation of metal nanoparticles or photosensitizers; (ii) increased self-cleaning application of superhydrophobic materials like graphenes and alkyl silanes; (iii) introducing photo- or electro thermal properties by adding graphene or metal thin films; (iv) stabilizing of electrostatic charges to maintain constant recharging^6^.

Metal-based antimicrobial technologies with silver, copper, titanium or zinc are well studied and applied in the functionalization of textiles^8,9^. However, they are suspected to cause skin sensitization and allergies^1^. The metal ion release is difficult to regulate and their fate in the environment is not always clear^10,11^. Therefore, safe and durable antiviral and antibacterial coatings for porous (e.g. textile) and non-porous materials (e.g. plastics and glass) would be highly needed.

Here we present a novel antiviral and antibacterial formulation provided by BASF corporation to treat porous surface materials (see Table 1). This proprietary formulation^12,13^ composed of alkyldimethylbenzylammonium chloride (ADBAC) as an active ingredient provides antiviral and antibacterial properties. In this study we developed a robust and easy-to-apply procedure to surface functionalize textiles, using a hospital curtain as a showcase. The developed coating strategy applying the investigated ADBAC coating aims for a rapid antiviral and antibacterial functionalization of a broad variety of textiles including the ones applied in the medical field such as hospital curtains and textiles in public transportation systems for single-use purposes. We assessed the mechanical stability and antiviral/antibacterial efficacy after coating, the functionality over time (shelf life of 6 months), and the potential coating release with artificial sweat. Additionally a KeratinoSens^®^ skin sensitization & irritation assay according to OECD guidelines No. 442D^14^ was conducted to elaborate on the potential effect of the coating on human skin.

**Table 1:** BASF coating, textiles, identifier, and description detail, specifications.

Identifier	Description	Supplier	Details
BASF antiviral coating liquid (BASF coating)	Liquid suspension containing, alkyldimethylbenzylammonium chloride (ADBAC), anionic polymeric binder, and surfactant. Dried coating by weight contains 50% ADBAC	BASF Corporation, Wyandotte MI, USA	BASF Coating-ES 9475, Lot: 60066425, patent application WO 2021/222748 A1 1–49 (2021)^12,13^
Reference curtain raw, water coated (RC WC)	Melt-blown PP textile, PEGATEX^®^SMS, 30 g/m^2^ antistatic, blue 401	PFNonwovens LLC, Hazleton PA, USA	Nonwoven fabric is suitable for the production of hygiene items, technical materials, packaging, and other products for which health safety is required
Reference curtain, coated (RC C)	0.5% ± 0.1% active BASF coating applied (N = 15)	Empa	Melt-blown PP textile, blue, in-house production is described in “Coating procedure (procedure, reproducibility, characterization)”
Hospital curtain, antimicrobial coated (HC AMC)	Melt-blown PP textile, disposable hospital curtain, pink	Wujiang Ruidi textile co., Ltd, Jiangsu, China	Melt-blown PP textile, pink, ISO 20743:2007 antiviral activity value > 4.4, TÜV SÜD Schweiz AG Art: 9139682, Test Nr: 922828-16-0707-01, SGS No: SHFD0100100058AN
Hospital curtain, antimicrobial and BASF antiviral coated (HC AM&VC)	0.5% ± 0.1% active BASF coating applied (N = 15)	Empa	Melt-blown PP textile, pink, in-house production is described in “Coating procedure (procedure, reproducibility, characterization)”

## Material and method

### Reference substances

#### Coating procedure (procedure, reproducibility, characterization)

The coating of textiles was optimized on a 2-roll padder type "HVF" coater (Werner Mathis AG, SN 20 589, Switzerland). During the optimization process of the coater, the following coating parameters were investigated (Table 2).

**Table 2:** Coating parameters evaluated for the establishing of a robust coating procedure yielding 0.5% dye weight of coating applying a 2-roll padder type "HVF" coater.

BASF in DI water (%)	Pressure between rolls (Bar)	Roll rotation speed (m/min)	Cycles (#)
5	1	0.2	1
7	2	1	2
10	4	3	3
20		5	4
30			5

**Figure 1:**
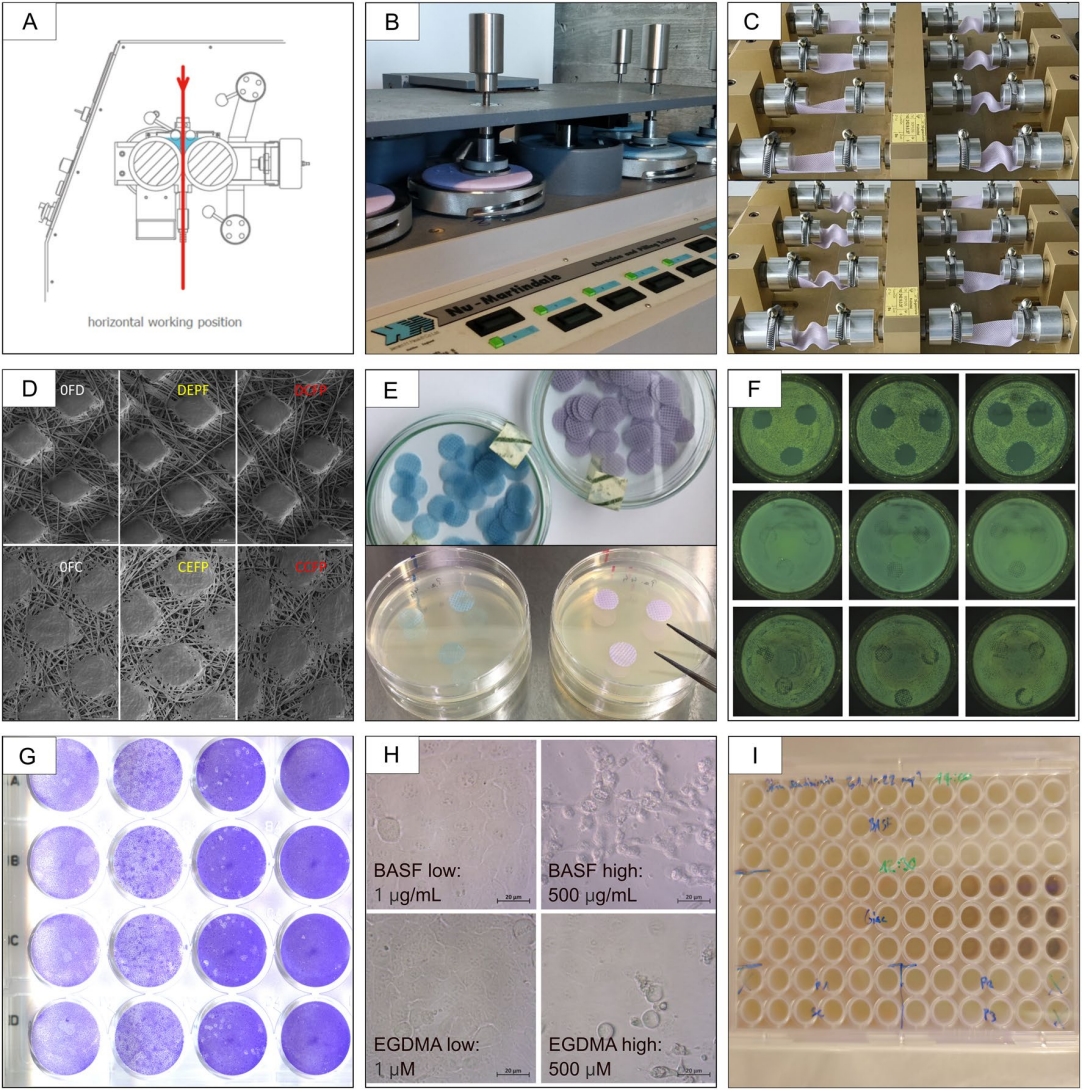
Graphical summary of the applied methods. (**A**) Coating of textiles was performed with a 2-roll padder type "HVF" coater. (**B**) The abrasion of textile samples was assessed by a Nu-Martindale abrasion and pilling tester instrument set to 9 kPa for 1000 cycles. (**C**) The textile durability was simulated on a fatigue instrument (EMPA) certified for EN ISO 7854:1997 testing with a 4 cm moving diameter and an amplitude of 8 cm. (**D**) SEM imaging of textiles was performed on an Axia ChemiSEM (Thermo Fisher, NL) using a secondary electron (SE) detector at an acceleration voltage of 10.00 kV. Individual images were collected (labeling: *white 0FD *control fatigue dummy, *0FC *control fatigue coated, *yellow DEPF *dummy external flexion point, *CEFP* coated external flexion point, *red DCFP* dummy central flexion point, *CCFP* coated central flexion point) and stitched using the software Maps 3.19 (Thermo Fisher, NL) to yield high-resolution overviews (spot area 4.0; magnification ×40). (**E**) Samples were prepared for the antibacterial touch test, punched out (diameter Ø 15 mm), and sterilized textiles. (**F**) Antibacterial efficacy against hospital pathogens including *A. baumanii*, *P. aeruginosa,* and *S. aureus* was measured both qualitatively with a touch test (4 h contacting time point shown) as well as quantitatively according to AATCC TM100. (**G**) The antiviral efficacy was assessed according to ISO 18184 comprising crystal violet staining of MHV-derived plaques within a L929 murine cell culture monolayer. (**H**) BASF coating (0.5–1000 µg mL^−1^) and ethylene glycol dimethylacrylate (EGDMA, 0.5–1000 µM) serving as skin sensitization positive control were added to KeratinoSens^®^ skin cells in serial dilutions. (**I**) KeratinoSens^®^ skin sensitization & irritation assay was performed according to OECD guidelines No. 442D.

After a screening process to identify the most promising ranges of parameters, coating trials were performed in triplicates (N = 3) to obtain a 0.5% weight coating (see Table S1). For coating, textile pieces were cut in pieces with the size of 15 cm × 4 cm (± 1 cm) and weighed before starting the coating procedure. Then, textile samples were soaked for 1 h in a BASF-DI water solution (10% v/v). After initial soaking, the textile went twice through the coater rolls, set to a pressure of 4 Bar. Finally, the textile samples were dried in a Mathis Labdryer oven (Werner Mathis AG, SN LTE113417, Switzerland) for 1 h at 80 °C, see Fig. 1 A. Subsequently, after coating the textile samples were weighted again to determine the coating uptake gravimetrically.

### Mechanical stability

#### Martindale abrasion

The abrasion of textile samples was assessed by the Nu-Martindale Abrasion and Pilling Tester instrument (James H. Heal & Co. Ltd., SN 403/96/2009, Halifax England) see Fig. 1B. Before the abrasion experiments textile samples were cut into 15 cm diameter pieces. The objective of the experiment was to observe damage to the coating and the fibers. The applied pressure to the textile samples was set to 9 kPa. A first trial using 10,000 cycles resulted in severe destruction of the textile, rendering gravimetry and SEM imaging impossible, therefore the number of cycles was reduced to 1000 in the following experiments. Sample preparation was performed in triplicates (N = 3). Gravimetrically the textile samples before and after Martindale abrasion were measured and the weight difference was calculated. Scanning electron microscope (SEM) imaging was performed to further distinguish between damage on the textile fibers and or damage attributed to the coating.

#### Textile durability

The textile durability simulation was performed with a fatigue instrument developed at Empa^15,16^ and certified for EN ISO 7854:1997 testing, see Fig. 1C. The instrument consists of a Sprecher S&S Schur motor with a 4 cm moving diameter and steel pistons with an amplitude of 8 cm. Textiles were cut to pieces with a length of 9.5 ± 0.5 cm and mounted to the steel pistons and folded with a frequency of 60 cycles min^-1^. Sample preparation was performed in triplicates (N = 3). According to the studies^17,18^, nurses in hospitals spend 37% of their time in contact with patients and perform 72 actions per hour. Action per hour accounts for the smallest countable tasks per nurse per time such as changing bed covers, moving patients, medical reporting, and patient medication to specify only a few tasks. They also estimate the presence of a nurse in a hospital ward to be 70 h a week. By multiplying an average nurse's action per hour by the weekly working hour and the percentage of patient contact a hospital curtain use scenario could be developed which could be mimicked by artificial aging. These figures may vary significantly depending on the regulations and practices of individual hospitals; however, they allow us to roughly estimate the number of weekly, monthly, and yearly folding an average hospital curtain may undergo (Simulation of a real-life hospital usage). Taking an optimistic approach, where all of the actions performed while in contact with the patients involve a curtain movement, the following number of cycles simulate the corresponding curtain usage: 3730 cycles (2 weeks), 22,378 cycles (3 months), and 492,316 cycles (5 years). After artificial aging the textiles were characterized both gravimetrically and with SEM imaging, see Fig. 1D.

### Functionality over time

#### Antibacterial assays

Two test methods for qualitative and quantitative evaluation were selected to assess the antibacterial activity of the functionalized textiles. In both assays, the Gram-positive *Staphylococcus aureus* MRSA (clinical patient isolate^19^, mecA resistant, similar to DSM11729), and Gram-negative *Pseudomonas aeruginosa* ATCC15442 and *Acinetobacter baumannii* ATCC19606 were used. The initial analysis was performed with the qualitative touch test to evaluate the antibacterial effectiveness of the textiles.

The fabrics were punched into circular pieces (diameter: Ø 15 mm) and sterilized by autoclaving at 121 °C for 20 min, see Fig. 1 E. All bacterial strains were revitalized from glycerol stocks stored at −80 °C by fractional streaking on Plate-Count agar (PC-agar) (Sigma Aldrich, 70152, Switzerland) and incubated at 37 °C overnight. One bacterial colony of each strain was picked from a PC-agar plate to inoculate 25 mL 30% Tryptic Soy Broth + 0.25% Glucose tryptic soy broth (TSB) (Sigma Aldrich, 22092, Switzerland) and further incubated at 37 °C at 160 rpm overnight. To determine bacterial growth, the turbidity (OD_600_) of the bacterial culture was measured at 600 nm using a Biophotometer Plus (Eppendorf, E-BP6132 00946, Germany). The overnight culture was diluted to an OD_600_ of 0.1 in 25 ml fresh TSB and cultivated for an additional 2 h until an exponential growth phase was reached. The bacterial culture was adjusted with phosphate-buffered saline (PBS) to a concentration of 10^5^ CFU mL^−1^, which corresponds to an OD_600_ of 0.01 for *P. aeruginosa* and *A. baumanni* and an OD_600 nm_ of 0.05 for *S. aureus*. 100 µL of this solution was applied with Easy Spiral Pro plater (Interscience, Ref 413000, France) to PC-agar plates. The agar plates were air-dried for 30 min to let the bacterial solution be absorbed by the agar. Sterilized fabrics were aseptically placed on the agar in triplicates for various time points (10 min, 1 h, 2 h, 4 h) and then removed. The plates were incubated at 37 °C for 18 h. The bacterial growth was compared with the modified and unmodified fabrics, see Fig. 1F. For quantitative measurement, the standard AATCC TM100^20^ was performed. Hence, the fabrics were cut into squares with a defined weight of 1.0 ± 0.1 g and subsequently inoculated with the bacteria strains mentioned above. The bacteria strains were contacted with the textile squares at 37 °C for a duration of 4 h. Afterwards the bacteria were retrieved with a PBS washing solution. The concentration of viable bacteria was determined by applying serial dilutions of this PBS washing solution to PC-agar plates. The antibacterial properties of textiles were determined by comparison of the recovered bacteria count from the textile sample at time points 0 h and 4 h, reported as a percent value.

The qualitative antibacterial tests comprise of agar plates seeded with a homogeneous bacteria lawn.

and contacted with textile pieces for a defined time (10 min & 4 h, shown within this manuscript, Fig. 3) and subsequently removed. An antibacterial effect can be nicely seen by the naked eye after incubation, on dark spots within the agar plates where bacteria growth is inhibited. Whereas the quantitative antibacterial tests assess the colony forming units CFU (single bacterial colonies on an agar plate) after textile incubation. The more cost-intensive quantitative antibacterial tests result is a countable reduction of bacteria after textile contacting time in comparison to control textile and non-contacting time (Fig. 4A, B).

#### Antiviral MHV according to ISO 18184

The antiviral capacity of the BASF-coated textiles was assessed according to ISO 18184:2019(E)^21^, see Fig. 1G. Below a brief description of the performed methodology is given. Virus strain (murine hepatitis virus MHV-A59, Coronavirus—Group 2, Biosafety Level: 2, ATCC^®^ VR1426^™^) and host cells (L929—murine subcutaneous connective tissue cell line for ISO 18184 plaque assay/17cl1—murine fibroblast cell line for viral propagation were a kind gift from Roland Züst, Laboratory Spiez, Switzerland)^22,23^. Within each plaque assay the wash-out of the virus immediately after the textile deposit (≤ 20 s), the wash-out of the virus after contact time (2 h), and a control test for verification of cytotoxicity of textile wash-out by cell sensitivity was assessed in independent triplicates (N = 3, n = 2). Cell cultures were propagated in minimal essential medium MEM (Sigma Aldrich, M2279, Switzerland) and wash-outs were performed with SCDLP solution (Huankai Microbial, 027010, China). 0.5% crystal violet (Sigma Aldrich, C3886-25G, USA) in 20% MeOH (Fluka, 65543, Switzerland) solution was applied to stain plaques within the cell culture monolayer. Finally for each ISO 18184 assay the antiviral activity value (Mv) − Mv = lg [mean (control immediately)] − lg [mean (antiviral 2 h)] and the precentral viral reduction (%VR) − %VR = [mean (antiviral 2 h)] × 100%/mean (control immediate) was calculated. The following combination (see Table 3) of control textile and antiviral textile were selected and assessed shortly after coating and 6 months post coating for shelf-life stability performance.

**Table 3:** Antiviral assessment assignment of sample coated (RC C and HC AM&VC) versus non-coated control (RC WC and HC AMC).

Assessment	Control textile	vs Antiviral textile
BASF 1	Reference curtain raw, water coated (RC WC)	Reference curtain, coated (RC C)
BASF 2	Hospital curtain, antimicrobial coated (HC AMC)	Hospital curtain, antiviral and antibacterial coated (HC AM&VC)
BASF 3	Reference curtain raw, water coated (RC WC)	Hospital curtain, antiviral and antibacterial coated (HC AM&VC)

For ISO 18184 plaque assay each run consists of non-coated control and an antiviral active sample. BASF 1 (reference curtain, blue) and BASF 2 (hospital curtain, pink) were assessed with and without BASF coating. In BASF 3 comparing the harshest conditions, a reference curtain (RC WC) was measured against an antiviral and antibacterial coated hospital curtain (HC AM&VC).

### Release of the active compound and cytotoxicity assessment

#### Coating leaching scenario with artificial sweat

To evaluate the extent of active release from the textiles coated with the BASF coating, the textiles were exposed to artificial sweat. In a typical experiment, 0.4 g BASF-coated textile (HC AM&VC) containing 0.5% BASF coating dry weight (DW) were immersed in 20 mL artificial sweat and kept for 2 h at 37 °C on a stationary shaker at 100 rpm^24,25^. Coating leaching experiments were performed in triplicated (N = 3) and compared to non-coated textiles (N = 3) treated under identical conditions. In a worst-case calculation assuming the entire coating would be dissolved within artificial sweat, a BASF coating concentration of 100 µg mL^−1^ could be expected. Samples were analyzed by UPLC–MS–MS (Waters, Acquity H Class-TQD system, Milford USA) and external multipoint calibration with reference compounds (alkyldimethylbenzylammonium chloride (ADBAC), R = C_12_H_25_, C_14_H_29_, C_16_H_31_; Limit of quantification LOQ < 100 ng mL^−1^) was performed.

#### Acute in vitro skin irritation and sensitization

To assess the most likely cause of hospital curtain contact with skin and sweat by nurses and patients, the in vitro skin toxicity of the BASF coating was analyzed. More specifically a KeratinoSens skin sensitization & irritation assay was performed according to OECD guidelines No. 442D^14^. This assay allows an estimation of the skin irritation (in vitro toxicity, see Fig. 1H) as well as the sensitization potential (see Fig. 1I). In short, the KeratinoSens skin cells (acCELLerate GmbH, RE242, Germany) were maintained in Dulbecco's Modified Eagle Medium (DMEM) low Glucose + 10% FBS + 1% l-Glutamine (DMEM complete) (Sigma Aldrich, D5546-500 mL, Switzerland) at 37 °C and 5% CO_2_. In 96 well plates, 10,000 cells per well were seeded and kept at 37 °C and 5% CO_2_ for 24 h. After a medium exchange with 150 µL DMEM complete, 50 µL of a serial sample dilution, positive control EGDMA (Sigma Aldrich, 33581-100 mL, Switzerland), Solvent control-DMEM complete containing 4% DMSO is added to the plate and kept for 2 days at 37 °C and 5% CO_2_. Hence the final concentrations of the 11 serial dilutions (N = 3, n = 3) were: sample: BASF coating (1–1000 µg mL^−1^), positive control: EGDMA (1–1000 µM) and artificial sweat (0.025–25%). After 2 days of contacting time, the viability of cells was assessed with Alamar Blue (Invitrogen, DAL1100, USA) 20µL for 4 h at excitation 540 nm/emission 590 nm. Afterward, plates were washed with 100 µL PBS (Sigma Aldrich, D8537—500 mL, Switzerland) and finally, a mixture of 50 µL One-Glo Reagent (Promega, E6120, USA) and 50 µL PBS was added to plates for 20 min at RT in darkness before luminescence was assessed with an integration time of 1 s/well. EC_1.5_ values of a dose-dependent increase in luciferase induction were calculated when viability values were ≥ 70% (according to OECD guidelines No. 442D)^14^.

The authors confirm that no human participants were involved in this study.

## Results and discussion

Quaternary ammonium compounds (QACs) are commonly used as antimicrobial agents in a variety of applications, including coatings for textiles. The use of QACs as coatings has been shown to provide effective antimicrobial protection against a range of microorganisms, including bacteria and fungi^26^. Several studies have investigated the efficacy of QACs as antimicrobial coatings on textiles. For example, Asri et al. developed a shape-adaptive, antibacterial coating of immobilized QACs tethered on hyperbranched polyurea, which was shown to provide effective antibacterial protection against both Gram-positive and Gram-negative bacteria^27^. Nevertheless, most of the reported QACs coating procedures use covalent strategies that are susceptible to the surface chemistry of the substrate and may require complex pretreatment steps. In this study, we report a facile QAC non-covalent functionalization method and use it for the coating of a hospital curtain case scenario. A coating deposited on porous surfaces, such as textiles, which do not interact with the surface carrier via covalent bonding can be performed cheaply and rapidly with already established industrial textile processing instruments and is therefore suited if antiviral and antibacterial are in urgent need. However, the intended use of non-covalently bound coating is in single-use functionalization and is therefore not washable without loss of antiviral and antibacterial efficacy. Applying a coating covalently to polypropylene textile is a more complex and therefore costlier method. Although covalently bound compounds could resist washing and are likely to be more durable, recycling is difficult, and in urgent pandemic scenarios is more difficult to scale up industrially. We investigated the stability of the coatings, their toxicological profile, and finally their antiviral and antibacterial performances.

### Coating procedure

**Figure 2:**
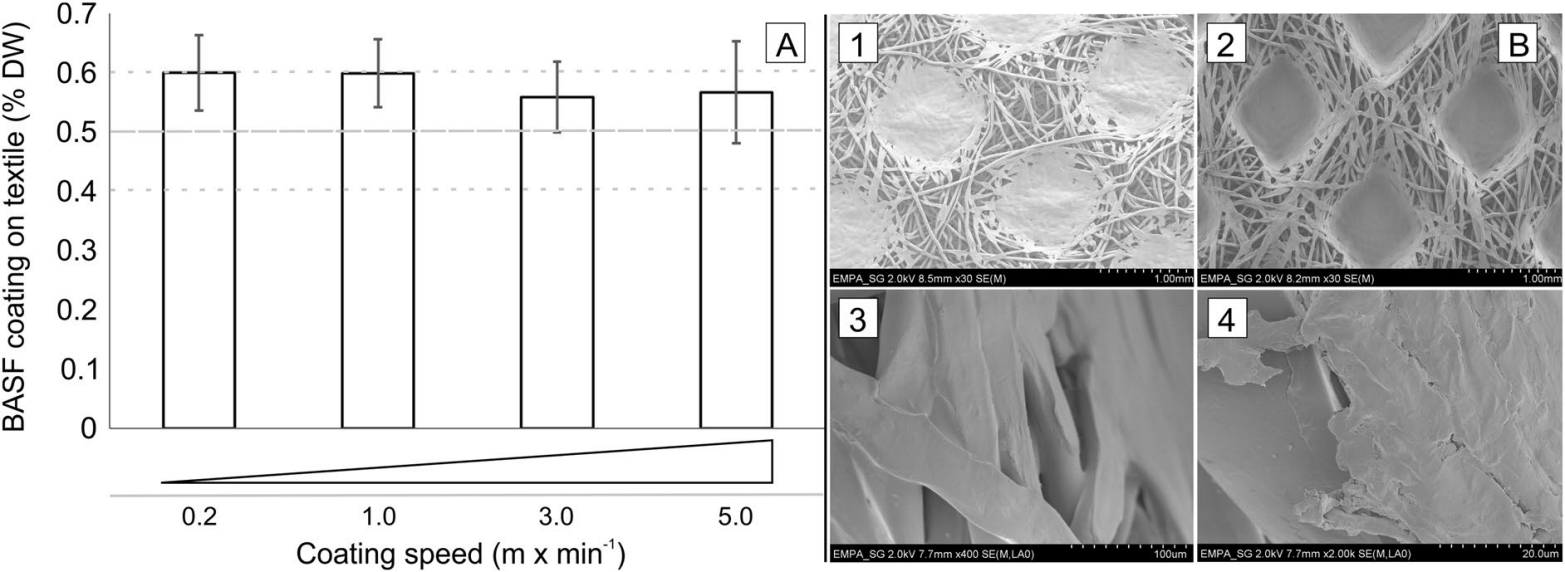
Reproducibility of the BASF coating procedure at coating speeds ranging from 0.2 to 5.0 m min^−1^ (**A**). The accuracy data were calculated (N = 6) after drying of the coated textile by subsequent gravimetrical determination of the BASF coating dry weight (DW) applied to the HC AMC textile. Further characterization of coated HC AMC textiles ((**B**), 2–4)) versus uncoated HC AMC textiles ((**B**), 1)) by SEM imaging revealed that the applied BASF coating at magnification of 20 µm up to 1000 µm was homogenously distributed. Hence, no BASF coating deposits were visual at the applied magnifications.

Aiming to develop an 'easy-to-apply' procedure to surface functionalize textiles with the antiviral and antibacterial coating, a 2-roll padder type "HVF" coater was used to non-covalently apply the BASF coating onto hospital curtain (HC AMC) as well as reference textile (RC WC). For establishing a suitable method the coating parameters (soaking: 1 h, Coating solution BASF liquid content in DI water: 7, 10, 15, 20 and 30% v/v, pressure: 1, 2 and 4 Bar, processing cycles: 1, 2, 4, 5 and 9×) were optimized whereas the drying conditions (time = 1 h, temperature = 80 °C) were kept constant. Details of the parameter optimization process are stated in the supplementary information (Table S1). The coating solution BASF liquid content in DI water proved to be an important factor aiming for a 0.5 ± 0.1% DW deposited on the textiles. Concentrations > 10, 15, 20, and 30% v/v) led to a textile deposition > 0.6% DW whereas 7% v/v yielded a textile deposition < 0.4% DW. The applied pressure between the 2-roll padders (1, 2, and 4 Bar) affected the uptake of the coating liquid before drying. Hence, as higher the pressure, the lower the liquid uptake by the textile leading to a dryer textile with evenly distributed liquid (Fig. S1). A pressure of 1 or 2 bar led to a wet textile with assumingly less homogeneous coating distribution on the textile after drying. The coating speed applied (0.2–5.0 m min^−1^) together with the number of processing cycles (1, 2, 4, 5, and 9×) had a negligible impact on the final BASF coating DW deposition onto the HC AMC textile. Targeting a BASF coating DW of 0.5 ± 0.1% deposited homogeneously onto the textile this coating method (coating parameters: soaking 1 h, coating solution BASF liquid 10% v/v in DI water, pressure 4 Bar, processing cycles 2×; drying conditions: time 1 h, temperature 80 °C) yielded the most accurate textile coating (Fig. 2A). An average BASF coating DW pickup by the HC AMC textile of 0.582% SD 0.027% was determined (N = 6, n = 3) by a limited variation on the coating speed applied (0.2–5.0 m min^−1^, see Fig. 2A). To further elaborate the homogeneity of the applied coating onto the hospital curtain HC AMC, scanning electron microscope (SEM) imaging with a magnification of 20 µm up to 1000 µm was conducted (Fig. 2B) showing no visible difference between uncoated HC AMC textile (B, 1) and coated HC AMC textiles (B, 2–4).

### Mechanical stability

The mechanical stability of the hospital curtain (HC AMC and HC AM&VC) and reference textile (RC WC and RC C) both coated and uncoated was assessed by two separate procedures. In this context, we tried to simulate the worst-case scenario where all the coating would be released. To do so, first, a Martindale abrasion experiment was conducted simulating the effect of curtain rubbing on each other e.g. during shipment of textiles or folding contact of curtains. Secondly, an artificial aging simulation corresponding to 2 weeks, 3 months, and 5 years of usage was performed on a fatigue instrument (Empa^15,16^) certified for EN ISO 7854:1997 testing. During both mechanical textile stability procedures applied, the textile integrity and structural textile fabric alterations were assessed gravimetrically for textile weight loss (severe alterations) and via SEM imaging for modest and structural fabric alterations. Additionally, SEM imaging was conducted to determine the fate of the non-covalently bound BASF coating within the applied textiles before and after treatment.

### Martindale abrasion

Martindale abrasion revealed only limited weight loss (1000 × cycles, 9 kPa, N = 3) of all assessed textiles (RC WC, RC C, HC AMC, and HC AM&VC) after treatment in comparison with fresh untreated textiles (see Table 4). However, with SEM imaging extensive damage to the textile structures down to single fibers was visible (Supplementary information Fig. S2). Although major textile and fiber damage was visible, it is speculated that this cannot be necessarily attributed to coating damage of equal severity, since SEM imaging revealed neither particulate solid nor crystal-like BASF coating deposits on coated textiles after Martindale abrasion.

**Table 4:** Abrasion assessment of textiles performed on a Nu-Martindale abrasion and pilling tester instrument.

Textiles	Weight before (g)	Weight after (g)	Weight loss (g)	Loss (%)	SD (%)
RC WC	0.565	0.556	0.009	1.56	2.79
RC C	0.527	0.527	0.000	-0.01	0.27
HC AMC	2.060	2.057	0.003	0.15	0.39
HC AM&VC	2.141	2.117	0.024	1.12	1.31

All experiments were performed in triplicates (N = 3) with equal textiles equipped on both panels of the Nu-Martindale instrument. Single measurements are stated in supplementary information in Table S2. The gravimetrically determined weight before & after as well as the weight loss (g and %) is stated.

Nu-Martindale abrasion, 1000 × cycles, 9 kPa pressure, N = 3.

### Durability

Compared to the Martindale abrasion (rather strong textile forces; worst case scenario) a more realistic hospital use scenario was applied during the durability artificial aging assessment. According to the literature^17,18^ nurses perform 72 times h^−1^ actions and spend 37% of their time with patients. In this scenario, it was assumed that the patient was always in contact with a nurse during working hours. Hence, if during each action the curtain is moved (folded, opened, or closed) once, the curtain undergoes 26.64 times h^−1^ movements (72 × 0.37 = 26.64). Including weekend work, 70 working hours per week were assumed. The artificial aging is calculated by multiplying the curtain h^−1^ movements (26.64 times h^−1^) with the working hours per week (70 h week^−1^) resulting in 1864.8 curtain movements per week. The fatigue instrument (Empa^15,16^) certified for EN ISO 7854:1997 testing, was set to 3730 cycles (3730 curtain movements equals 2 weeks), 22,378 cycles (22,378 curtain movements equals 3 months) and 492,316 cycles (curtain movements equals 5 years) for artificial aging.

During the hospital use durability scenario, in terms of gravimetrical loss difference (≤ 0.5%, see Table 5) and via SEM imaging (visual inspection) no major alteration of the textile fabrics and its applied coating was reported. These indicated the stability of the non-covalently bound BASF coating on both textiles for up to 5 years.

**Table 5:** Durability determination after artificial hospital curtain aging scenario.

Textiles	% loss after 2 weeks	SD %	% loss after 3 months	SD %	% loss after 5 years	SD %
RC WC	−0.27	0.20	−0.16	0.11	0.35	0.25
RC C	−0.07	0.28	−0.21	0.11	0.41	0.56
HC AMC	−0.05	0.03	0.00	0.00	−0.06	0.07
HC AM&VC	−0.16	0.06	0.10	0.05	−0.04	0.05

The weight loss of textile (%), as well as the standard deviation (%), is stated after textile treatment with the fatigue instrument (Empa^15,16^) set to 3730 cycles (equals 2 weeks), 22,378 cycles (equals 3 months) and 492,316 cycles (equals 5 years).

Durability after artificial aging 2 weeks to 5 years, amplitude 8 cm, frequency 60 cycles min^−1^, N = 3.

### Functionality over time

#### Antibacterial assay

The antibacterial activity of the modified textiles HC AMC and HC AM&VC was evaluated qualitatively using a touch test using RC WC and RC C as controls. The obtained results were classified according to the following categories: Excellent, maximum 1–5 CFU per plate; Good, maximum 5–20 CFU; Moderate, 20+ CFU; Poor, no significant difference to control.

**Figure 3:**
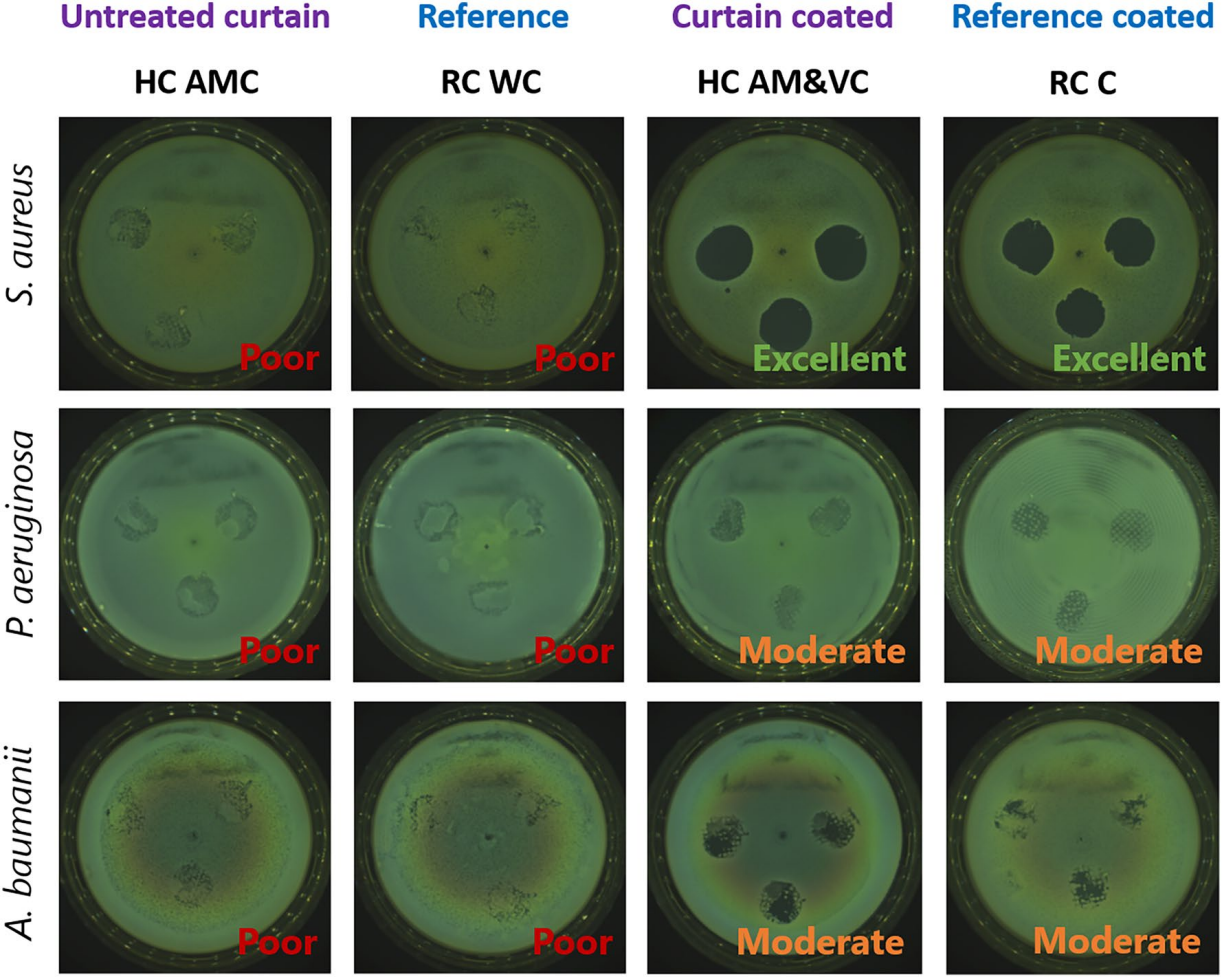
Results obtained by the touch test. Reference curtain (RC WC); reference curtain, BASF coated (RC C); hospital curtain antiviral and antibacterial coated (HC AMC) and hospital curtain antiviral and antibacterial BASF coated (HC AM&VC) were tested against *S. aureus*, *P. aeruginosa,* and *A. baumanii* after 4 h of contacting time.

The commercially available hospital curtain, antiviral and antibacterial coated (HC AMC) and the uncoated reference curtain (RC WC) did not lead to any visible inhibition in bacterial growth of *S. aureus*, *P. aeruginosa,* and *A. baumanii* (see Fig. 3), whereas the in-house BASF coated reference curtain (RC C) and the hospital curtain BASF coated (HC AM&VC) showed excellent antibacterial efficacy against *S. aureus* and moderate antibacterial activity against *P. aeruginosa* and *A. baumanii*. These results indicate that the commercially available hospital curtain antiviral and antibacterial coated (HC AMC) does not have antibacterial activity during the 4 h contacting time. To investigate the stability of the antibacterial activity of the modified fabrics, the samples aged for 6 months were also evaluated by the touch test. All samples after 6 months of storage showed the same antibacterial profile as the initial ones. This first investigation resulted in a possible effect for rapid contact killings through the BASF coating since reproducible effects could already be shown after 10 min of treatment (data shown in the supplementary information Fig. S3).

**Figure 4:**
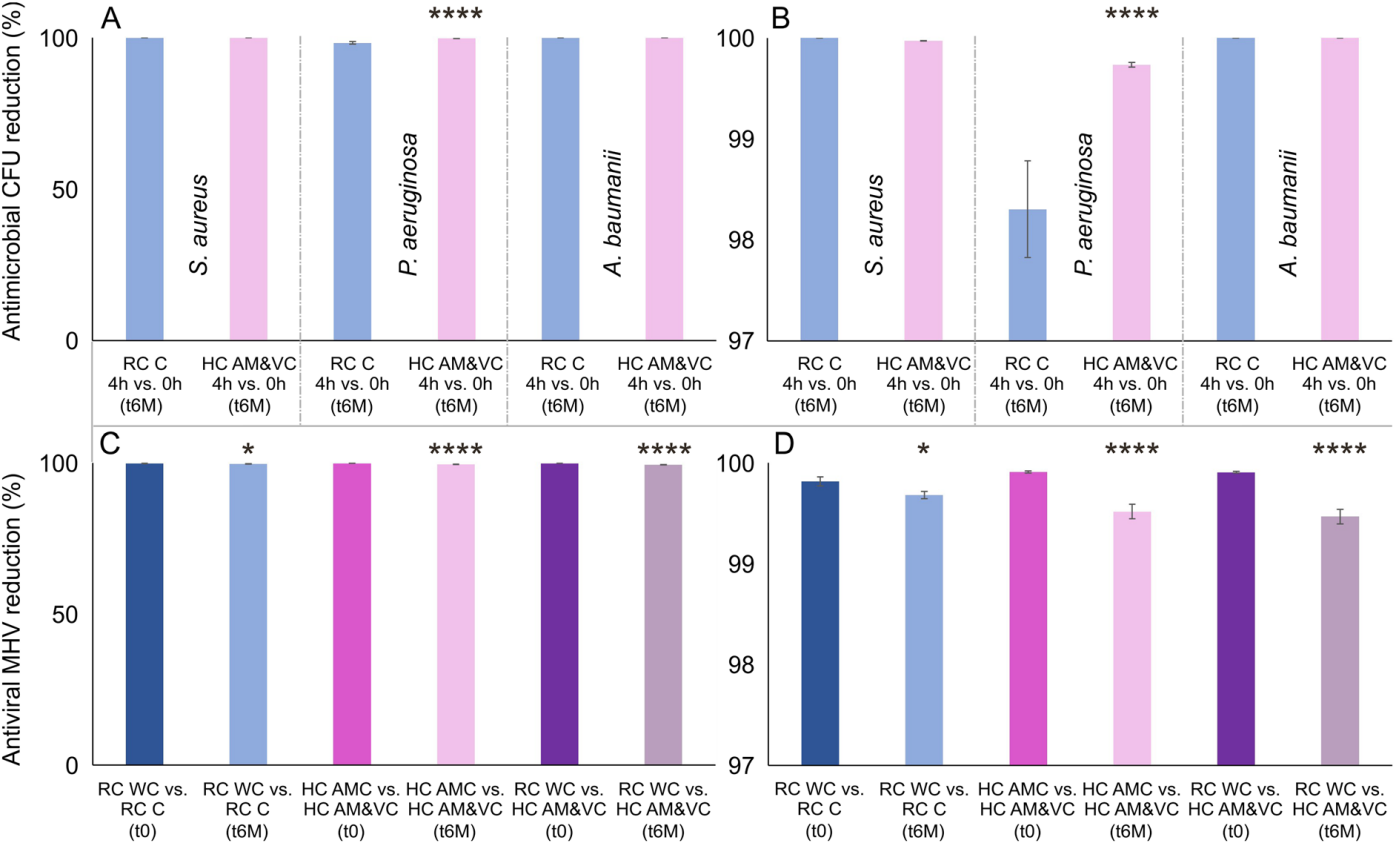
Antibacterial (**A,B**) and antiviral (**C,D**) properties of BASF coated textiles RC C and HC AM&VC. The reduction in colony forming unit (CFU) (%) after 4 h interaction with the coated RC C and HC AM&VC textiles after 6 months of storage (t6M) is illustrated (**A,B**). The hospital microbial strains *S. aureus*, *P. aeruginosa* & *A. baumanii* were assessed (N = 3, n = 2) and the % CFU reduction was calculated by comparison of CFU t0 0 h versus CFU t4 after 4 h interacting time. The antiviral assessment was performed with freshly BASF coated (t0) and 6-month-aged (t6M) textiles according to ISO 18184^21^ with murine hepatitis virus (MHV) (**C,D**). The % viral reduction (N = 3, n = 2) was calculated by comparison of uncoated textile (RC WC and HC AMC) versus BASF coated textile (RC C and HC AM&VC). The scale bar of (**A,C**) is indicated from 0 to 100% reduction and for better visibility zoomed from 97 to 100% in image (**B,D**). Two-way ANOVA analysis comparing textile samples (HC AMC & HC AM&VC) with control textiles (RC C & RC WC) regarding antiviral (**A,B**) and antibacterial (**C,D**) efficacy is indicated in the figure by stars (*p ≤ 0.05; ****p ≤ 0.0001). Full statistical two-way ANOVA analysis is stated in the supplementary information in Tables S3 and S4.

The quantitative antibacterial efficacy was measured with all textiles after 6 months of shelf live storage according to AATCC TM100-2019^20^ (see Fig. 4A, B), using *S. aureus*, *P. aeruginosa* & *A. baumanii*. The limit of detection (LoD) for antibacterial efficacy is < 100 colony forming units (CFU) at the lowest 10^0^ assay dilution. The cell viability was assessed based on the reduction of the CFU after 4 h incubation with the samples compared to 0 h (Fig. 4A, B). For better visualization, in Fig. 4A the results are shown with a scale bar of 0–100% CFU reduction and additionally, a zoom from 97 to 100% CFU reduction in Fig. 4B is given. The quantitative antibacterial efficacy measured by AATCC TM100-2019 was in agreement with that observed in the qualitative touch test. Within 4 h interaction, the RC C and HC AM&VC led to a CFU reduction of 99.99 ± 0.00% (RC C) and 99.97 ± 0.00% (HC AM&VC), respectively, for *S. aureus*, 98.30 ± 0.48% (RC C) and 99.73 ± 0.02% (HC AM&VC), respectively, *P. aeruginosa*, and 100 ± 0.00% for both textiles for *A. baumanii*. Within the assessed time range of 6 months an effective antibacterial activity was achieved by both BASF-coated textiles for the applied hospital strains.

### Antiviral MHV according to ISO 18184

The antiviral capacity of textiles freshly (t0) BASF coated and after shelf live storage (t6M) was performed according to ISO-18184^21^ over 2 h with a murine hepatitis virus (MHV) system. As part of each standardized assay (N = 3, n = 2) the plaque forming units concentration (pfu/mL), the antiviral activity value (Mv), and finally the antiviral reduction in % were calculated. A table summarizing all gained antiviral results is provided in the supplementary information Table S5.

Overall, the BASF coating showed an excellent antiviral capacity with an average antiviral % reduction of 99.65 ± 0.21% (N = 18, n = 2) with the highest reduction of 99.91 ± 0.01% achieved with HC AMC vs. HC AM&VC (t0, N = 3, n = 2) and the lowest reduction of 99.47 ± 0.07% with RC WC vs. HC AM&VC (t6M, N = 3, n = 2). Regarding to shelf live stability only a minor reduction of antiviral capacity was observed on aging 99.55 ± 0.11% (t6M mean, N = 9, n = 2) when compared with freshly coated textiles 99.88 ± 0.05% (t0 mean, N = 9, n = 2) (see Fig. 4C, D). The antiviral activity was calculated for each condition according to ISO-18184^21^ stated in the supplementary information Table S5. Furthermore, the preliminary ISO-18184 assay data allowed us to calculate that the contacting time of 2h with non-coated textiles alone reduces the viral content on an average of 64.38 ± 14.14% (N = 18, n = 2) (see Table S5). When coated and non-coated textiles both contacted for 2 h were compared an average reduction of 98.70 ± 0.98% (N = 18, n = 2) was achieved by the coated textile compared to the non-coated textile (see Table S5).

### Coating leaching scenario with artificial sweat

Release of active compound into artificial sweat was investigated by UPLC–MS–MS detection in triplicates (N = 3, n = 3). As mentioned previously, a worst-case scenario calculation assuming the entire coating would be dissolved within the artificial sweat, a BASF coating DW concentration of 100 µg mL^−1^ is expected after extraction. Since the BASF coating DW contains 50% active compound (quaternary ammonia) within its formulation the DW concentration correlated to 50 µg mL^−1^ active compound. UPLC–MS–MS analytics of the BASF coated textile (HC AM&VC & RC C) extracts after 2 h at 37 °C revealed a BASF active compound concentration on average of 24 ± 5 µg mL^−1^. As expected, no active compound (quaternary ammonia) was detected for non-BASF coated textiles (HC AMC & RC WC) above the LOQ of < 100 ng mL^−1^.

### Acute in vitro skin toxicity & skin sensitization

**Figure 5:**
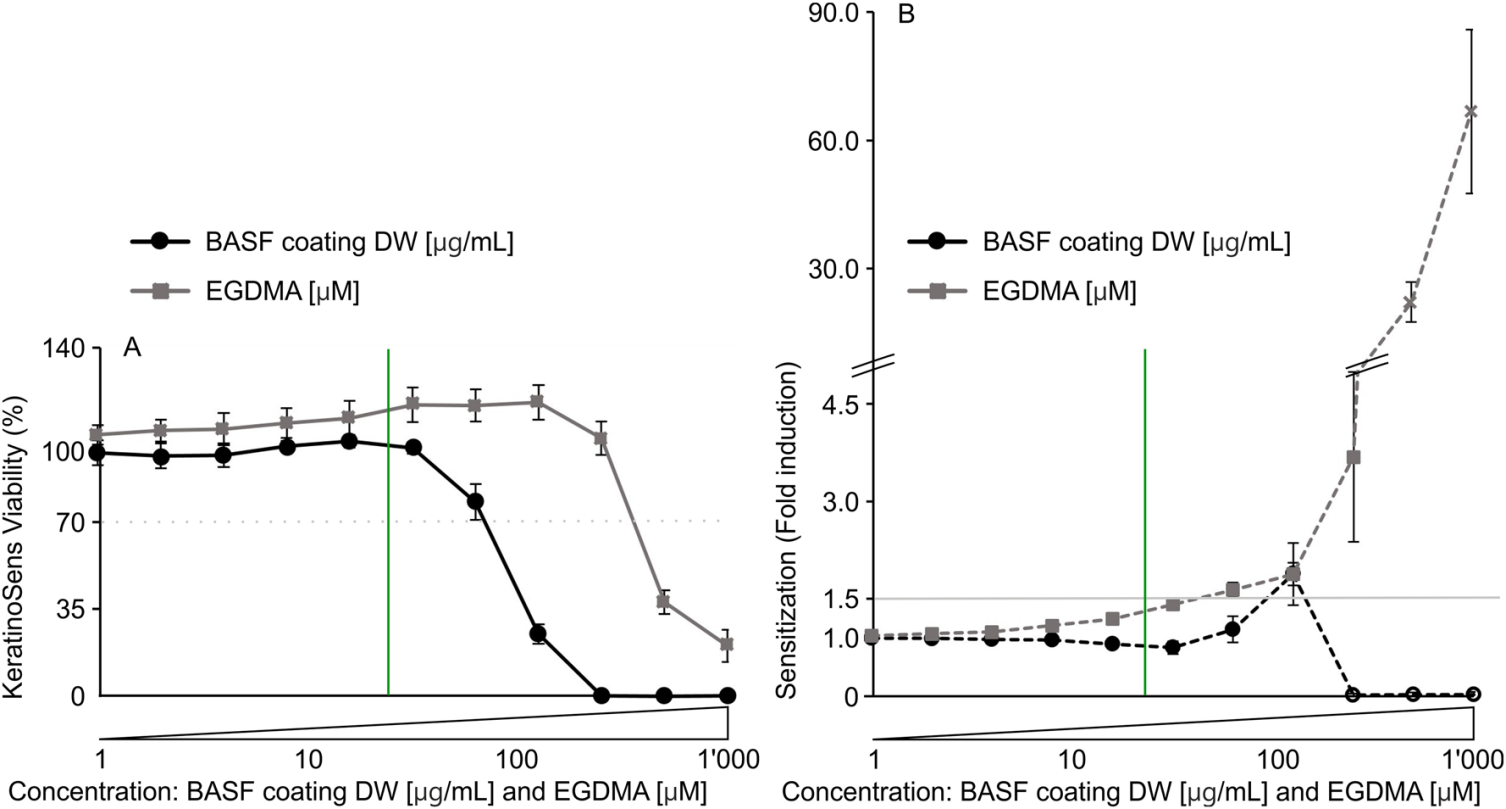
In vitro skin irritation and skin sensitization potential of the BASF coating. In serial dilution ranging from 1 to 1000 µg mL^−1^ (BASF coating DW) and positive control and known skin sensitizer ethylene glycol dimethylacrylate (EGDMA) 1–1000 µM were assessed with the KeratinoSens^®^ skin sensitization & irritation assay performed according to OECD guidelines No. 442D^14^. (**A**) Skin irritation is shown, black circles indicate the assessed BASF coating, and grey rectangular the EGDMA concentrations. Values below 70% viability, see grey dotted line (**A**) indicate the irritation concentration. The green line indicates the BASF active compound concentration (24 ± 5 µg mL^−1^) analytically detected after artificial sweat extraction. (**B**) Skin sensitization is shown. A 1.5-fold induction (EC_1.5_) compared to the solvent control (solid grey line, (**B**)) indicates a sensitization if ≥ 70% viability (**A**) is given at identical concentrations. The Y-axis is divided, allowing us to illustrate the sensitization course of both substances: BASF coating (0–2 fold induction) and EGDMA (0–50 fold induction). Experiments were conducted in triplicates (N = 3, n = 3).

To assess the acute skin toxicity and skin sensitization potential of the BASF coating a KeratinoSens^®^ skin sensitization & irritation assay performed according to OECD guidelines No. 442D^14^ was performed (Fig. 5). The BASF coating was analyzed in serial dilutions ranging from 1 to 1000 µg mL^−1^ BASF coating dry weight (DW) corresponding to 0.5–500 µg mL^−1^ active compound. A concentration of 1000 µg mL^−1^ exceeds the worst-case scenario concentration (100 µg mL^−1^ BASF coating DW) by 10 times at which the entire coating would be released by contact with artificial sweat. Included within this study is EGDMA in serial dilutions ranging from 1 to 1000 µM which is a well described^14,28^ weak skin sensitizer for comparison. The IC_50_ (50% viable cells) is reported at a concentration of > 500 μM for skin irritation and an EC_1.5_ (Luciferase induction of 1.5 compared to the control) range of 5–125 μM for EGDMA^28^. Within this study, an IC_50_ concentration of 453 μM and an EC_1.5_ value of 43 μM was measured for EGDMA, showing the accuracy of the assay performed (Fig. 5). The BASF coating revealed an IC_50_ concentration of 95 µg mL^−1^ whereas the EC_1.5_ concentration could not be determined according to OECD guidelines No. 442D^14^ acceptance criteria (EC_1.5_ below 70% viability) leading to the conclusion that the BASF coating is a non-sensitizing-agent (see Fig. 5). Although within this study the BASF coating containing alkyldimethylbenzylammonium chloride (ADBAC) compounds was not categorized as skin sensitizing agent, in the literature irritant, sensitizing, and adjuvant properties of quaternary ammonium compounds have been reported^29,30^. Benzalkonium chloride (BAC) and benzethonium chloride showed in vitro strong skin cytotoxicity and histological skin damage^29^. A BAC IC_50_ of 1.2 µg mL^−1^ for adult normal human epidermal keratinocytes (NHEK), 3.0 µg mL^−1^ for neonatal normal human epidermal keratinocytes (NHDF), 1.2 µg mL^−1^ for human leukemia monocytic cell line (THP-1) and 0.5 µg mL^−1^ for promyelocytic leukemia cell line (HL-60) was reported^30^. The acute in vitro skin toxicity & skin sensitization results highlight that leaching of coating from the textile to body fluids such as sweat is likely. However, when leaching of BASF coating occurs the assessed worst-case scenario investigated would not result in skin irritation or sensitization in the applied textile coating concentration. Furthermore, the study shows as expected that the coating is not washable and therefore is intended for single-use antiviral and antibacterial functionalization of textiles.

## Conclusion and outlook

Within this study, it was demonstrated that the BASF coating rendered the surfaces of textiles both antibacterial and antiviral. Analysis of textiles revealed that the non-covalently applied BASF coating containing alkyldimethylbenzylammonium chloride as an active ingredient was homogeneously distributed (0.5 ± 0.1% coating DW) in both polypropylene non-woven hospital curtains and reference textiles. Furthermore, the simulated use study showed the structural fabric and coating integrity was maintained for up to 5 years. This was verified by applying Martindale abrasion and durability testing under simulated curtain use conditions that were analyzed by SEM and gravimetrical analysis. The high durability of the BASF coating provides an effective advantage over other less durable antiviral and antibacterial technologies for textiles. This proof of concept demonstrated that the BASF coating could be used to make porous materials such as textiles antiviral and antibacterial. After aging for 6 months, the BASF-coated hospital curtain maintained a high antibacterial (t6M = *S. aureus*: 99.97 ± 0.00%; *P. aeruginosa*: 99.73 ± 0.02%; *A. baumanii*: 100 ± 0.00%) and antiviral (t0 = 99.88 ± 0.05%, t6M = 99.55 ± 0.11%, N = 9, n = 2) efficacy. During a leaching study where the BASF-coated hospital curtain was contacted with artificial sweat, an active compound concentration of 24 ± 5 µg mL^−1^ was recovered and analytically quantified. The acute KeratinoSens^®^ skin sensitization & irritation assay revealed an IC_50_ concentration of 95 µg mL^−1^ and is categorized as a non-sensitizing agent according to OECD guidelines No. 442D^14^ criteria. Although the BASF coating is not washable, with its intended use as a coating for single-use textile functionalization the applied BASF coating concentration is not likely to cause skin irritation & sensitization as the worst-case leaching study indicates. The recovered active compound concentration (24 ± 5 µg mL^−1^) is below the assessed IC_50_ concentration. The BASF coating provided good shelf life and mechanical stability, considering the activity in the coating was non-covalently bonded to the textile. This coating allows for treated textiles to be safely stored and then later converted to hospital curtains, while maintaining antiviral and antibacterial activity. Such a coating allows fast and efficient functionalization of hospital curtains based on need. Continued research involving safe antibacterial and antiviral coatings for surfaces is needed to help prevent the spread of pathogens limiting future epidemics and pandemics.

### Supplementary Information

Supplementary Information.

## Data Availability

All data generated or analyzed during this study are included in this published article and its supplementary information file.
