# Supplementary material for: Quaternary ammonium-based coating of textiles is effective against bacteria and viruses with a low risk to human health
Source: Sci Rep. 2023 Nov 23;13:20556. doi: 10.1038/s41598-023-47707-3 (PMC10667359; doi:10.1038/s41598-023-47707-3)
Supplement: Supplementary file 1 — Supplementary Information. [file 41598_2023_47707_MOESM1_ESM.docx]

**Quaternary ammonium-based coating of textiles is effective against bacteria and viruses with a low risk to human health.**

*Philipp Meier^1^, Pietro Clement^1^, Stefanie Altenried^2^, Giacomo Reina^1^, Qun Ren^2^, Roland Züst^3^, Olivier Enger^4^, Francis Ming Hei Choi^5^, Nikolaus Nestle^6^, Ted Deisenroth^7^, Peter Neubauer^8^, Peter Wick^1^**

*^1^ Particles-Biology Interactions Laboratory, Empa – Swiss Federal Laboratories for Materials Science and Technology, St. Gallen 9014, Switzerland*

*^2^ Biointerfaces Laboratory, Empa – Swiss Federal Laboratories for Materials Science and Technology, St. Gallen 9014, Switzerland*

*^3^ Federal Office for Civil Protection FOCP, Spiez Laboratory, Spiez 3700, Switzerland*

*^4^ Technology Scouting & Incubation, BASF Schweiz AG, Basel 4005, Switzerland*

*^5^ BASF Corporation, 1609 Biddle Avenue, Wyandotte 48192 MI, USA*

*^6^ BASF SE, Carl-Bosch-Strasse 38, Ludwigshafen am Rhein 67056, Germany*

*^7^ Formulation Research, BASF Corporation, 500 White Plains Road, New York 10591 Tarrytown, USA*

*^8^ Chair of Bioprocess Engineering, Institute for Biotechnology, TU Berlin, 13355 Berlin, Germany*

*Contact information corresponding author:

Peter Wick – Particles-Biology Interactions Laboratory, Empa - Swiss Federal Laboratories for Materials Science and Technology, St. Gallen 9014, Switzerland; orcid.org/0000-0002-0079-4344; Phone: +41 58 765 7684; Email: peter.wick@empa.ch

**Supplementary information:**

Figures: 3

Tables: 5

**Developing a smart and fast textile functionalization coating to fight against bacteria and viruses.**

- **Coating procedure**

Methodology described in chapter:

2.2 Coating procedure (procedure, reproducibility, characterization)

**Table S1:** Details of the parameter applied during the optimization process. The coating of textiles was optimized on a 2-roll padder type "HVF" coater (Werner Mathis AG, SN 20 589, Switzerland). The following coating parameters were investigated.

| **BASF liquid content in DI water [% v/v]** | **Processing cycles [x]** | **Pressure [Bar]** | **Speed [m/min]** | **Soaking [h]** | **BASF coating, Replicate 1 [% DW]** | **BASF coating, Replicate 2 [% DW]** | **BASF coating, Replicate 3 [% DW]** | **Average** | **SD** |
| --- | --- | --- | --- | --- | --- | --- | --- | --- | --- |
| 10 | 2 | 1 | 0.2 | no | 0.410 | 0.375 | 0.408 | 0.398 | 0.016 |
| 10 | 2 | 1 | 0.2 | no | 0.555 | 0.525 | 0.537 | 0.539 | 0.012 |
| 10 | 2 | 1 | 0.2 | no | 0.673 | 0.589 | 0.740 | 0.668 | 0.062 |
| 10 | 2 | 1 | 0.2 | no | 0.354 | 0.368 | 0.373 | 0.365 | 0.008 |
| 10 | 2 | 1 | 0.2 | no | 0.481 | 0.531 | 0.563 | 0.525 | 0.034 |
| 10 | 2 | 1 | 0.2 | no | 0.451 | 0.448 | 0.472 | 0.457 | 0.011 |
| 10 | 2 | 1 | 2.0 | no | 0.471 | 0.460 | 0.435 | 0.455 | 0.015 |
| 10 | 2 | 1 | 2.0 | no | 0.413 | 0.452 | 0.412 | 0.426 | 0.019 |
| 10 | 2 | 4 | 0.2 | 1 | 0.596 | 0.663 | 0.654 | 0.638 | 0.030 |
| 10 | 2 | 4 | 0.2 | 1 | 0.641 | 0.521 | 0.527 | 0.563 | 0.055 |
| 10 | 2 | 4 | 1.0 | 1 | 0.634 | 0.647 | 0.500 | 0.594 | 0.066 |
| 10 | 2 | 4 | 1.0 | 1 | 0.560 | 0.623 | 0.635 | 0.606 | 0.033 |
| 10 | 2 | 4 | 3.0 | 1 | 0.549 | 0.507 | 0.608 | 0.555 | 0.042 |
| 10 | 2 | 4 | 3.0 | 1 | 0.656 | 0.526 | 0.511 | 0.564 | 0.065 |
| 10 | 2 | 4 | 5.0 | 1 | 0.547 | 0.612 | 0.522 | 0.560 | 0.038 |
| 10 | 2 | 4 | 5.0 | 1 | 0.520 | 0.482 | 0.720 | 0.574 | 0.104 |


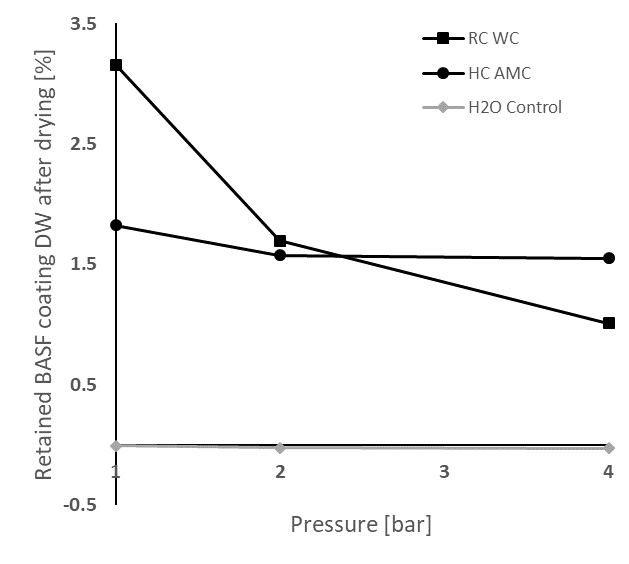


**Figure S1:** BASF coating dry-weight (DW) uptake by the textiles (RC C and HC AMC) in dependence of the applied pressure aiming for an evenly distributed liquid coating.

- **Mechanical stability - Martindale abrasion**

Methodology described in chapter:

2.3 Mechanical stability (Martindale abrasion)


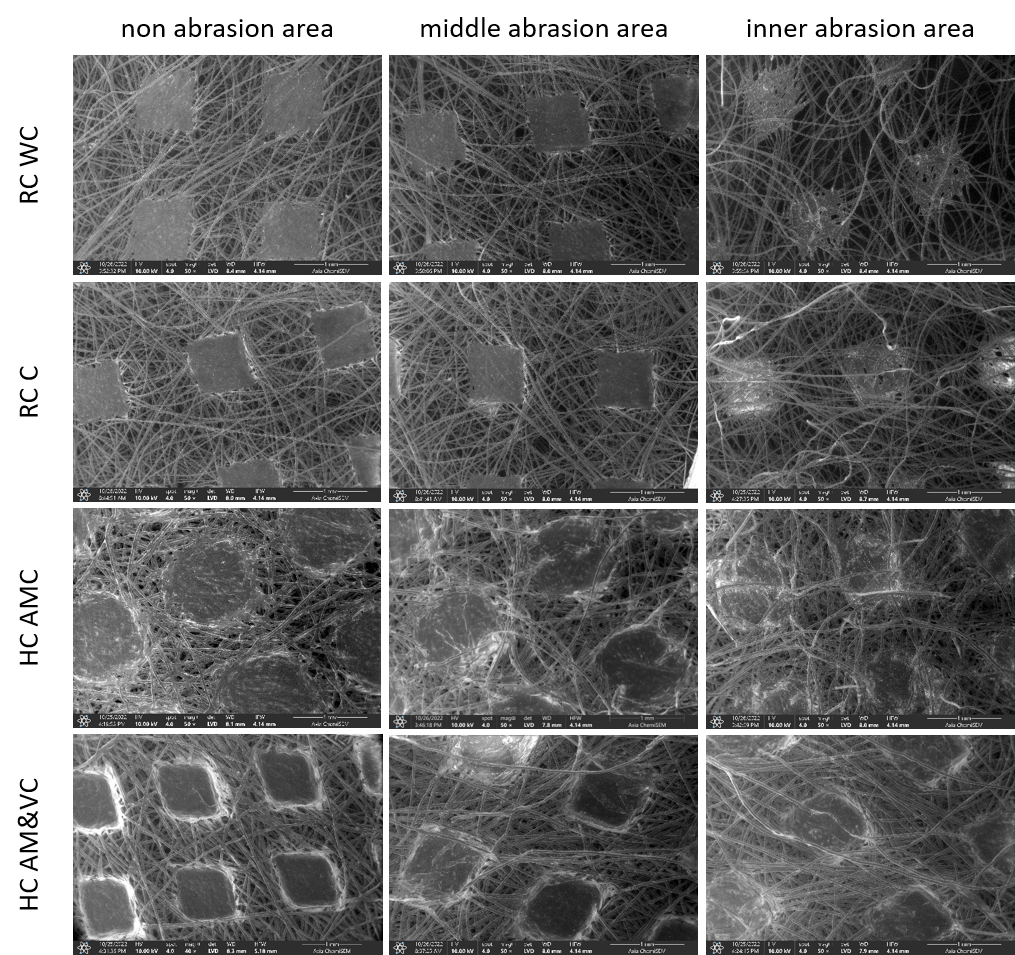


**Figure S2:** SEM imaging of the textile (RC WC, RC C, HC AMC and HC AM&VC) structure after Martindale abrasion (1000x cycles, 9 kPa). Extensive damage of the textile structure down to the single fibers was visible for all textiles at the inner abrasion area and partly at the middle abrasion area when compared to the non-abrasion area. However, no BASF coating release (particle deposits or crystals like structures) was observed in all BASF coated textiles (RC C and HC AM&VC) after abrasion.

**Table S2:** Single measurements of abrasion assessment of textiles performed on a Nu-Martindale Abrasion and Pilling Tester 309 instrument.

| Textiles | Weight before [g] | Weight after [g] | Weight loss [g] | Loss [%] | SD [%] |
| --- | --- | --- | --- | --- | --- |
| RC WC, sample 1 | 0.566 | 0.540 | -0.027 | 0.05 | 4.68 |
| RC WC, sample 2 | 0.574 | 0.578 | 0.004 | -0.01 | -0.70 |
| RC WC, sample 3 | 0.555 | 0.551 | -0.004 | 0.01 | 0.72 |
| RC C, sample 1 | 0.541 | 0.540 | -0.002 | 0.00 | 0.30 |
| RC C, sample 2 | 0.558 | 0.559 | 0.001 | 0.00 | -0.18 |
| RC C, sample 3 | 0.483 | 0.484 | 0.001 | 0.00 | -0.17 |
| HC AMC, sample 1 | 2.097 | 2.096 | -0.002 | 0.00 | 0.07 |
| HC AMC, sample 2 | 2.073 | 2.061 | -0.012 | 0.01 | 0.58 |
| HC AMC, sample 3 | 2.009 | 2.013 | 0.004 | 0.00 | -0.20 |
| HC AM&VC, sample 1 | 2.209 | 2.201 | -0.008 | 0.00 | 0.35 |
| HC AM&VC, sample 2 | 2.134 | 2.125 | -0.009 | 0.00 | 0.42 |
| HC AM&VC, sample 3 | 2.079 | 2.024 | -0.055 | 0.03 | 2.65 |

- **Antibacterial assay**

Results described in chapter:

3.3 Functionality over time - Antibacterial assay.


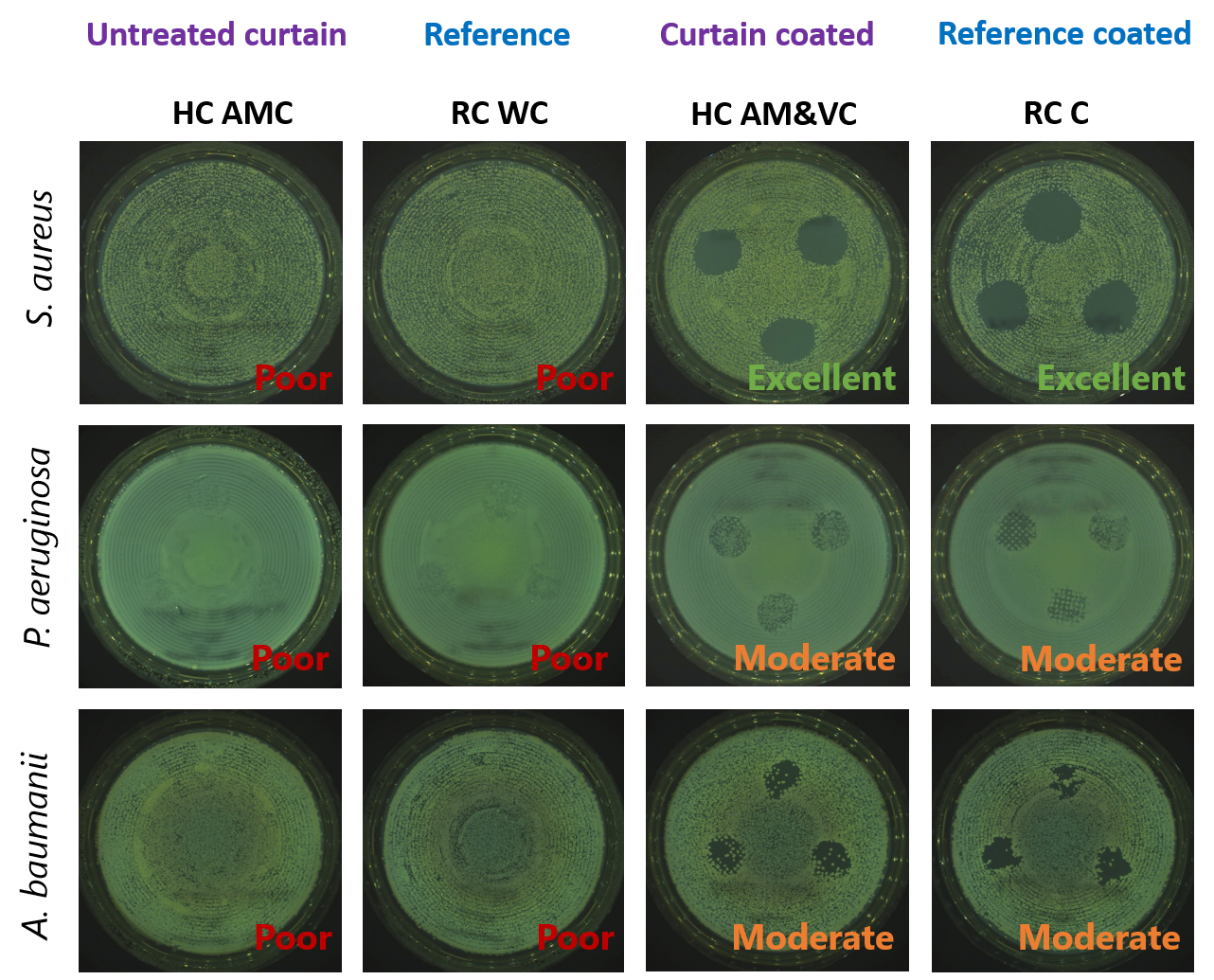


**Figure S3:** Results obtained by the touch test. Reference curtain (RC WC); reference curtain, BASF coated (RC C); Hospital curtain antiviral and antibacterial coated (HC AMC) and hospital curtain antiviral and antibacterial BASF coated (HC AM&VC) were tested against *S. aureus*, *P. aeruginosa* and *A. baumanii* after 10 min. of contacting time.

- **Statistical analysis of antiviral and antibacterial efficacy (Figure 4) of BASF-coated textile fresh (t0) and aged (t6M) vs uncoated textile**

**Table S3:** Statistical analysis antibacterial efficacy: Figure 4 A&B

| Table Analyzed | Data 1 |  |  |  |  |
| --- | --- | --- | --- | --- | --- |
|  |  |  |  |  |  |
| Two-way ANOVA | Ordinary |  |  |  |  |
| Alpha | 0.05 |  |  |  |  |
|  |  |  |  |  |  |
| Source of Variation | % of total variation | P value | P value summary | Significant? |  |
| Interaction | 28.44 | <0.0001 | **** | Yes |  |
| Row Factor | 13.43 | 0.0003 | *** | Yes |  |
| Column Factor | 51.83 | <0.0001 | **** | Yes |  |
|  |  |  |  |  |  |
| ANOVA table | SS | DF | MS | F (DFn, DFd) | P value |
| Interaction | 2.086 | 2 | 1.043 | F (2, 12) = 27.08 | P<0.0001 |
| Row Factor | 0.9851 | 1 | 0.9851 | F (1, 12) = 25.58 | P=0.0003 |
| Column Factor | 3.801 | 2 | 1.900 | F (2, 12) = 49.35 | P<0.0001 |
| Residual | 0.4621 | 12 | 0.03851 |  |  |
|  |  |  |  |  |  |
|  |  |  |  |  |  |

| Compare cell means regardless of rows and columns |  |  |  |  |  |  |  |  |
| --- | --- | --- | --- | --- | --- | --- | --- | --- |
|  |  |  |  |  |  |  |  |  |
| Number of families | 1 |  |  |  |  |  |  |  |
| Number of comparisons per family | 15 |  |  |  |  |  |  |  |
| Alpha | 0.05 |  |  |  |  |  |  |  |
|  |  |  |  |  |  |  |  |  |
| Tukey's multiple comparisons test | Mean Diff. | 95.00% CI of diff. | Significant? | Summary | Adjusted P Value |  |  |  |
|  |  |  |  |  |  |  |  |  |
| RC C:S. aureus vs. RC C:P. aeruginosa | 1.696 | 1.158 to 2.234 | Yes | **** | <0.0001 |  |  |  |
| RC C:S. aureus vs. RC C:A. baumanii | -0.001740 | -0.5399 to 0.5364 | No | ns | >0.9999 |  |  |  |
| RC C:S. aureus vs. HC AM&VC:S. aureus | 0.02689 | -0.5113 to 0.5651 | No | ns | >0.9999 |  |  |  |
| RC C:S. aureus vs. HC AM&VC:P. aeruginosa | 0.2653 | -0.2729 to 0.8034 | No | ns | 0.5816 |  |  |  |
| RC C:S. aureus vs. HC AM&VC:A. baumanii | -0.001740 | -0.5399 to 0.5364 | No | ns | >0.9999 |  |  |  |
| RC C:P. aeruginosa vs. RC C:A. baumanii | -1.698 | -2.236 to -1.159 | Yes | **** | <0.0001 |  |  |  |
| RC C:P. aeruginosa vs. HC AM&VC:S. aureus | -1.669 | -2.207 to -1.131 | Yes | **** | <0.0001 |  |  |  |
| RC C:P. aeruginosa vs. HC AM&VC:P. aeruginosa | -1.431 | -1.969 to -0.8924 | Yes | **** | <0.0001 |  |  |  |
| RC C:P. aeruginosa vs. HC AM&VC:A. baumanii | -1.698 | -2.236 to -1.159 | Yes | **** | <0.0001 |  |  |  |
| RC C:A. baumanii vs. HC AM&VC:S. aureus | 0.02863 | -0.5095 to 0.5668 | No | ns | >0.9999 |  |  |  |
| RC C:A. baumanii vs. HC AM&VC:P. aeruginosa | 0.2670 | -0.2712 to 0.8052 | No | ns | 0.5753 |  |  |  |
| RC C:A. baumanii vs. HC AM&VC:A. baumanii | 0.000 | -0.5382 to 0.5382 | No | ns | >0.9999 |  |  |  |
| HC AM&VC:S. aureus vs. HC AM&VC:P. aeruginosa | 0.2384 | -0.2998 to 0.7765 | No | ns | 0.6778 |  |  |  |
| HC AM&VC:S. aureus vs. HC AM&VC:A. baumanii | -0.02863 | -0.5668 to 0.5095 | No | ns | >0.9999 |  |  |  |
| HC AM&VC:P. aeruginosa vs. HC AM&VC:A. baumanii | -0.2670 | -0.8052 to 0.2712 | No | ns | 0.5753 |  |  |  |
|  |  |  |  |  |  |  |  |  |
|  |  |  |  |  |  |  |  |  |
| Test details | Mean 1 | Mean 2 | Mean Diff. | SE of diff. | N1 | N2 | q | DF |
|  |  |  |  |  |  |  |  |  |
| RC C:S. aureus vs. RC C:P. aeruginosa | 100.0 | 98.30 | 1.696 | 0.1602 | 1 | 1 | 14.97 | 12.00 |
| RC C:S. aureus vs. RC C:A. baumanii | 100.0 | 100.0 | -0.001740 | 0.1602 | 1 | 1 | 0.01536 | 12.00 |
| RC C:S. aureus vs. HC AM&VC:S. aureus | 100.0 | 99.97 | 0.02689 | 0.1602 | 1 | 1 | 0.2373 | 12.00 |
| RC C:S. aureus vs. HC AM&VC:P. aeruginosa | 100.0 | 99.73 | 0.2653 | 0.1602 | 1 | 1 | 2.341 | 12.00 |
| RC C:S. aureus vs. HC AM&VC:A. baumanii | 100.0 | 100.0 | -0.001740 | 0.1602 | 1 | 1 | 0.01536 | 12.00 |
| RC C:P. aeruginosa vs. RC C:A. baumanii | 98.30 | 100.0 | -1.698 | 0.1602 | 1 | 1 | 14.98 | 12.00 |
| RC C:P. aeruginosa vs. HC AM&VC:S. aureus | 98.30 | 99.97 | -1.669 | 0.1602 | 1 | 1 | 14.73 | 12.00 |
| RC C:P. aeruginosa vs. HC AM&VC:P. aeruginosa | 98.30 | 99.73 | -1.431 | 0.1602 | 1 | 1 | 12.63 | 12.00 |
| RC C:P. aeruginosa vs. HC AM&VC:A. baumanii | 98.30 | 100.0 | -1.698 | 0.1602 | 1 | 1 | 14.98 | 12.00 |
| RC C:A. baumanii vs. HC AM&VC:S. aureus | 100.0 | 99.97 | 0.02863 | 0.1602 | 1 | 1 | 0.2527 | 12.00 |
| RC C:A. baumanii vs. HC AM&VC:P. aeruginosa | 100.0 | 99.73 | 0.2670 | 0.1602 | 1 | 1 | 2.357 | 12.00 |
| RC C:A. baumanii vs. HC AM&VC:A. baumanii | 100.0 | 100.0 | 0.000 | 0.1602 | 1 | 1 | 0.000 | 12.00 |
| HC AM&VC:S. aureus vs. HC AM&VC:P. aeruginosa | 99.97 | 99.73 | 0.2384 | 0.1602 | 1 | 1 | 2.104 | 12.00 |
| HC AM&VC:S. aureus vs. HC AM&VC:A. baumanii | 99.97 | 100.0 | -0.02863 | 0.1602 | 1 | 1 | 0.2527 | 12.00 |
| HC AM&VC:P. aeruginosa vs. HC AM&VC:A. baumanii | 99.73 | 100.0 | -0.2670 | 0.1602 | 1 | 1 | 2.357 | 12.00 |
|  |  |  |  |  |  |  |  |  |

**Table S4:** Statistical analysis of antiviral efficacy: Figure 4 C&D

| Table Analyzed | | Data 2 |  | |  | |  |  |
| --- | --- | --- | --- | --- | --- | --- | --- | --- |
|  | |  |  | |  | |  |  |
| Two-way ANOVA | | Ordinary |  | |  | |  |  |
| Alpha | | 0.05 |  | |  | |  |  |
|  | |  |  | |  | |  |  |
| Source of Variation | | % of total variation | P value | | P value summary | | Significant? |  |
| Interaction | | 13.75 | 0.0003 | | *** | | Yes |  |
| Row Factor | | 79.46 | <0.0001 | | **** | | Yes |  |
| Column Factor | | 1.958 | 0.1302 | | ns | | No |  |
|  | |  |  | |  | |  |  |
| ANOVA table | | SS | DF | | MS | | F (DFn, DFd) | P value |
| Interaction | | 0.08085 | 2 | | 0.04043 | | F (2, 12) = 17.05 | P=0.0003 |
| Row Factor | | 0.4673 | 1 | | 0.4673 | | F (1, 12) = 197.1 | P<0.0001 |
| Column Factor | | 0.01151 | 2 | | 0.005757 | | F (2, 12) = 2.428 | P=0.1302 |
| Residual | | 0.02845 | 12 | | 0.002371 | |  |  |
|  | |  |  | |  | |  |  |
|  |  | |  |  | |  | |  |
|  |  | |  |  | |  | |  |

| Compare cell means regardless of rows and columns |  |  |  |  |  |  |  |  |
| --- | --- | --- | --- | --- | --- | --- | --- | --- |
|  |  |  |  |  |  |  |  |  |
| Number of families | 1 |  |  |  |  |  |  |  |
| Number of comparisons per family | 15 |  |  |  |  |  |  |  |
| Alpha | 0.05 |  |  |  |  |  |  |  |
|  |  |  |  |  |  |  |  |  |
| Tukey's multiple comparisons test | Mean Diff. | 95.00% CI of diff. | Significant? | Summary | Adjusted P Value |  |  |  |
|  |  |  |  |  |  |  |  |  |
| t0:RC DC vs. RC C vs. t0:HC AMC vs. HC AM&VC | -0.09381 | -0.2274 to 0.03973 | No | ns | 0.2438 |  |  |  |
| t0:RC DC vs. RC C vs. t0:RC DC vs. HC AM&VC | -0.09079 | -0.2243 to 0.04276 | No | ns | 0.2716 |  |  |  |
| t0:RC DC vs. RC C vs. t6M:RC DC vs. RC C | 0.1347 | 0.001135 to 0.2682 | Yes | * | 0.0477 |  |  |  |
| t0:RC DC vs. RC C vs. t6M:HC AMC vs. HC AM&VC | 0.2985 | 0.1649 to 0.4320 | Yes | **** | <0.0001 |  |  |  |
| t0:RC DC vs. RC C vs. t6M:RC DC vs. HC AM&VC | 0.3490 | 0.2155 to 0.4825 | Yes | **** | <0.0001 |  |  |  |
| t0:HC AMC vs. HC AM&VC vs. t0:RC DC vs. HC AM&VC | 0.003025 | -0.1305 to 0.1366 | No | ns | >0.9999 |  |  |  |
| t0:HC AMC vs. HC AM&VC vs. t6M:RC DC vs. RC C | 0.2285 | 0.09494 to 0.3620 | Yes | *** | 0.0010 |  |  |  |
| t0:HC AMC vs. HC AM&VC vs. t6M:HC AMC vs. HC AM&VC | 0.3923 | 0.2587 to 0.5258 | Yes | **** | <0.0001 |  |  |  |
| t0:HC AMC vs. HC AM&VC vs. t6M:RC DC vs. HC AM&VC | 0.4428 | 0.3093 to 0.5764 | Yes | **** | <0.0001 |  |  |  |
| t0:RC DC vs. HC AM&VC vs. t6M:RC DC vs. RC C | 0.2255 | 0.09192 to 0.3590 | Yes | ** | 0.0011 |  |  |  |
| t0:RC DC vs. HC AM&VC vs. t6M:HC AMC vs. HC AM&VC | 0.3892 | 0.2557 to 0.5228 | Yes | **** | <0.0001 |  |  |  |
| t0:RC DC vs. HC AM&VC vs. t6M:RC DC vs. HC AM&VC | 0.4398 | 0.3062 to 0.5733 | Yes | **** | <0.0001 |  |  |  |
| t6M:RC DC vs. RC C vs. t6M:HC AMC vs. HC AM&VC | 0.1638 | 0.03024 to 0.2973 | Yes | * | 0.0139 |  |  |  |
| t6M:RC DC vs. RC C vs. t6M:RC DC vs. HC AM&VC | 0.2143 | 0.08079 to 0.3479 | Yes | ** | 0.0017 |  |  |  |
| t6M:HC AMC vs. HC AM&VC vs. t6M:RC DC vs. HC AM&VC | 0.05054 | -0.08300 to 0.1841 | No | ns | 0.7944 |  |  |  |
|  |  |  |  |  |  |  |  |  |
|  |  |  |  |  |  |  |  |  |
| Test details | Mean 1 | Mean 2 | Mean Diff. | SE of diff. | N1 | N2 | q | DF |
|  |  |  |  |  |  |  |  |  |
| t0:RC DC vs. RC C vs. t0:HC AMC vs. HC AM&VC | 99.81 | 99.91 | -0.09381 | 0.03976 | 1 | 1 | 3.337 | 12.00 |
| t0:RC DC vs. RC C vs. t0:RC DC vs. HC AM&VC | 99.81 | 99.91 | -0.09079 | 0.03976 | 1 | 1 | 3.229 | 12.00 |
| t0:RC DC vs. RC C vs. t6M:RC DC vs. RC C | 99.81 | 99.68 | 0.1347 | 0.03976 | 1 | 1 | 4.791 | 12.00 |
| t0:RC DC vs. RC C vs. t6M:HC AMC vs. HC AM&VC | 99.81 | 99.52 | 0.2985 | 0.03976 | 1 | 1 | 10.62 | 12.00 |
| t0:RC DC vs. RC C vs. t6M:RC DC vs. HC AM&VC | 99.81 | 99.47 | 0.3490 | 0.03976 | 1 | 1 | 12.41 | 12.00 |
| t0:HC AMC vs. HC AM&VC vs. t0:RC DC vs. HC AM&VC | 99.91 | 99.91 | 0.003025 | 0.03976 | 1 | 1 | 0.1076 | 12.00 |
| t0:HC AMC vs. HC AM&VC vs. t6M:RC DC vs. RC C | 99.91 | 99.68 | 0.2285 | 0.03976 | 1 | 1 | 8.128 | 12.00 |
| t0:HC AMC vs. HC AM&VC vs. t6M:HC AMC vs. HC AM&VC | 99.91 | 99.52 | 0.3923 | 0.03976 | 1 | 1 | 13.95 | 12.00 |
| t0:HC AMC vs. HC AM&VC vs. t6M:RC DC vs. HC AM&VC | 99.91 | 99.47 | 0.4428 | 0.03976 | 1 | 1 | 15.75 | 12.00 |
| t0:RC DC vs. HC AM&VC vs. t6M:RC DC vs. RC C | 99.91 | 99.68 | 0.2255 | 0.03976 | 1 | 1 | 8.020 | 12.00 |
| t0:RC DC vs. HC AM&VC vs. t6M:HC AMC vs. HC AM&VC | 99.91 | 99.52 | 0.3892 | 0.03976 | 1 | 1 | 13.85 | 12.00 |
| t0:RC DC vs. HC AM&VC vs. t6M:RC DC vs. HC AM&VC | 99.91 | 99.47 | 0.4398 | 0.03976 | 1 | 1 | 15.64 | 12.00 |
| t6M:RC DC vs. RC C vs. t6M:HC AMC vs. HC AM&VC | 99.68 | 99.52 | 0.1638 | 0.03976 | 1 | 1 | 5.826 | 12.00 |
| t6M:RC DC vs. RC C vs. t6M:RC DC vs. HC AM&VC | 99.68 | 99.47 | 0.2143 | 0.03976 | 1 | 1 | 7.624 | 12.00 |
| t6M:HC AMC vs. HC AM&VC vs. t6M:RC DC vs. HC AM&VC | 99.52 | 99.47 | 0.05054 | 0.03976 | 1 | 1 | 1.798 | 12.00 |
|  |  |  |  |  |  |  |  |  |
|  |  |  |  |  |  |  |  |  |

- **Antiviral MHV according to ISO 18184**

Methodology described in chapter:

2.4 Functionality over time (Antiviral MHV according to ISO 18184)

**Table S5:** Antiviral capacity of textiles freshly (t0) BASF coated and after shelf live storage (t6M) was performed according to ISO-18184 over 2h with a murine hepatitis virus (MHV) system. For each standardized assay (N=3, n=2) the plaque forming units concentration (pfu/mL), the antiviral activity value (Mv), and finally the antiviral reduction in % were calculated. Additionally, the acceptance criteria are stated.

|  |  | **Virus reduction (%)** | | | |
| --- | --- | --- | --- | --- | --- |
|  | **Mv = lg (mean [control immediate]) - lg (mean [antiviral 2h])** | Only textile (cont. text.) 0-2h | Antiviral textile (BASF coat) 2h vs. cont. textile (non-coated) 2h | Combined antiviral textile (BASF coat) 2h v.s. IW cont. textile (non-coated) 0h |  |
| BASF 1 | **2.71** | 56.54 | 99.55 | **99.80** |  |
| BASF 2 | **2.38** | 51.94 | 99.13 | **99.58** |  |
| BASF 3 | **3.46** | 50.81 | 99.93 | **99.97** |  |
| 6M BASF 1 | **2.29** | 73.70 | 98.03 | **99.48** |  |
| 6M BASF 2 | **2.47** | 86.92 | 97.42 | **99.66** |  |
| 6M BASF 3 | **2.21** | 66.37 | 98.16 | **99.38** |  |
|  |  |  |  |  |  |
|  | **Acceptance criteria** | | |  | |
|  | lg (pfu/ml control) - lg (pfu/ml antiviral) ≤ 0.5 | M = lg (mean [control immediate]) - lg (mean [control]) ≤ 1.0 | Acceptance |  | |
| BASF 1 | -0.19 | 0.36 | passed |  | |
| BASF 2 | 0.21 | 0.32 | passed |  | |
| BASF 3 | 0.30 | 0.31 | passed |  | |
| 6M BASF 1 | 0.04 | 0.58 | passed |  | |
| 6M BASF 2 | 0.00 | 0.88 | passed |  | |
| 6M BASF 3 | 0.04 | 0.47 | passed |  | |
|  |  |  |  |  | |
|  | **Virus concentrations (pfu/mL)** | | |  | |
|  | Immediate wash-out, cont. textile | 2h textile contacting time, cont. textile | 2h textile contacting time, antiviral textile |  | |
| BASF 1 | 67111 | 29167 | 132 |  | |
| BASF 2 | 131667 | 63278 | 549 |  | |
| BASF 3 | 127500 | 62722 | 44 |  | |
| 6M BASF 1 | 37833 | 9950 | 196 |  | |
| 6M BASF 2 | 25167 | 3292 | 85 |  | |
| 6M BASF 3 | 22750 | 7650 | 141 |  | |
